# Genetic studies in *Drosophila* and humans support a model for the concerted function of *CISD2*, *PPT1* and *CLN3* in disease

**DOI:** 10.1242/bio.20147559

**Published:** 2014-04-04

**Authors:** Melanie A. Jones, Sami Amr, Aerial Ferebee, Phung Huynh, Jill A. Rosenfeld, Michael F. Miles, Andrew G. Davies, Christopher A. Korey, John M. Warrick, Rita Shiang, Sarah H. Elsea, Santhosh Girirajan, Mike Grotewiel

**Affiliations:** 1 Department of Human and Molecular Genetics, Virginia Commonwealth University, Richmond, VA 23298, USA; 2 Molecular Biology and Genetics Program, Virginia Commonwealth University, Richmond, VA 23298, USA; 3 Signature Genomic Laboratories, Spokane, WA 99207, USA; 4 Department of Pharmacology and Toxicology, Virginia Commonwealth University, Richmond, VA 23298, USA; 5 Department of Biology, College of Charleston, Charleston, SC 29401, USA; 6 Department of Biology, University of Richmond, Richmond, VA 23173, USA; 7 Department of Molecular and Human Genetics, Baylor College of Medicine, Houston, TX 77030, USA; 8 Department of Biochemistry and Molecular Biology, Pennsylvania State University, University Park, PA 16802, USA; 9 Department of Anthropology, Pennsylvania State University, University Park, PA 16802, USA

**Keywords:** RNA interference, Neurodegeneration, Genetic modifiers, Wolfram syndrome, Copy number variants, Lysosomal storage disease, Gene network

## Abstract

Wolfram syndrome (WFS) is a progressive neurodegenerative disease characterized by diabetes insipidus, diabetes mellitus, optic atrophy, and deafness. WFS1 and WFS2 are caused by recessive mutations in the genes *Wolfram Syndrome 1* (*WFS1*) and *CDGSH iron sulfur domain 2* (*CISD2*), respectively. To explore the function of *CISD2*, we performed genetic studies in flies with altered expression of its *Drosophila* orthologue, *cisd2*. Surprisingly, flies with strong ubiquitous RNAi-mediated knockdown of *cisd2* had no obvious signs of altered life span, stress resistance, locomotor behavior or several other phenotypes. We subsequently found in a targeted genetic screen, however, that altered function of *cisd2* modified the effects of overexpressing the fly orthologues of two lysosomal storage disease genes, *palmitoyl-protein thioesterase 1* (*PPT1* in humans, *Ppt1* in flies) and *ceroid-lipofuscinosis*, *neuronal 3* (*CLN3* in humans, *cln3* in flies), on eye morphology in flies. We also found that *cln3* modified the effects of overexpressing *Ppt1* in the eye and that overexpression of *cln3* interacted with a loss of function mutation in *cisd2* to disrupt locomotor ability in flies. Follow-up multi-species bioinformatic analyses suggested that a gene network centered on *CISD2*, *PPT1* and *CLN3* might impact disease through altered carbohydrate metabolism, protein folding and endopeptidase activity. Human genetic studies indicated that copy number variants (duplications and deletions) including *CLN3*, and possibly another gene in the *CISD2/PPT1/CLN3* network, are over-represented in individuals with developmental delay. Our studies indicate that *cisd2*, *Ppt1* and *cln3* function in concert in flies, suggesting that *CISD2*, *PPT1* and *CLN3* might also function coordinately in humans. Further, our studies raise the possibility that WFS2 and some lysosomal storage disorders might be influenced by common mechanisms and that the underlying genes might have previously unappreciated effects on developmental delay.

## INTRODUCTION

Wolfram syndrome (WFS) is an autosomal recessive neurodegenerative disease that affects 1 in 770,000 people in the United Kingdom (Barrett et al., 1995). Affected individuals present with diabetes insipidus, diabetes mellitus, optic atrophy and deafness (Wolfram, 1938). Other features of this syndrome include psychiatric illness (Strom et al., 1998) and renal-tract abnormalities (Barrett et al., 1995). Patients usually die within the third decade of life due to respiratory failure associated with brainstem atrophy (Scolding et al., 1996). Mutations in two genes, *WFS1* (Strom et al., 1998) and *CISD2* (Amr et al., 2007) are known to cause WFS1 and WFS2, respectively. *WFS1* encodes wolframin, a transmembrane protein that localizes to the endoplasmic reticulum (ER). Wolframin is important for intracellular calcium homeostasis and is a downstream component of IRE1 and PERK signaling in the unfolded protein response (Osman et al., 2003; Fonseca et al., 2005).

*CISD2*, the second WFS locus, was more recently identified (Amr et al., 2007). A homozygous splice site mutation in *CISD2* that eliminates the full-length transcript was found in three Jordanian families with WFS2 (Amr et al., 2007). *CISD2* encodes a protein with one predicted transmembrane domain and one predicted iron–sulfur domain (Amr et al., 2007; Wiley et al., 2007). Like wolframin, the *CISD2* gene product localizes to the ER (Amr et al., 2007), but whether *CISD2* is involved in regulation of the unfolded protein response has not been addressed. *Cisd2* knockout mice exhibit neurodegeneration along with shortened lifespan (Chen et al., 2009). These mice also have mitochondrial degeneration (Chen et al., 2009), suggesting that *CISD2* is important for mitochondrial integrity and that mitochondrial dysfunction might contribute to the pathology of WFS2. Despite these and other advances in understanding *CISD2*, its function has not been fully resolved. Here, we describe genetic studies in the fruit fly, *Drosophila melanogaster*, and human genetic studies that provide insight into the function of *CISD2*. Our data support a gene network model in which *CISD2* might function in concert with *PPT1*, *CLN3* and several other genes under normal or possibly pathological states.

## RESULTS

### Identification and RNAi-mediated knockdown of *Drosophila cisd2*

**Fig. 1.**
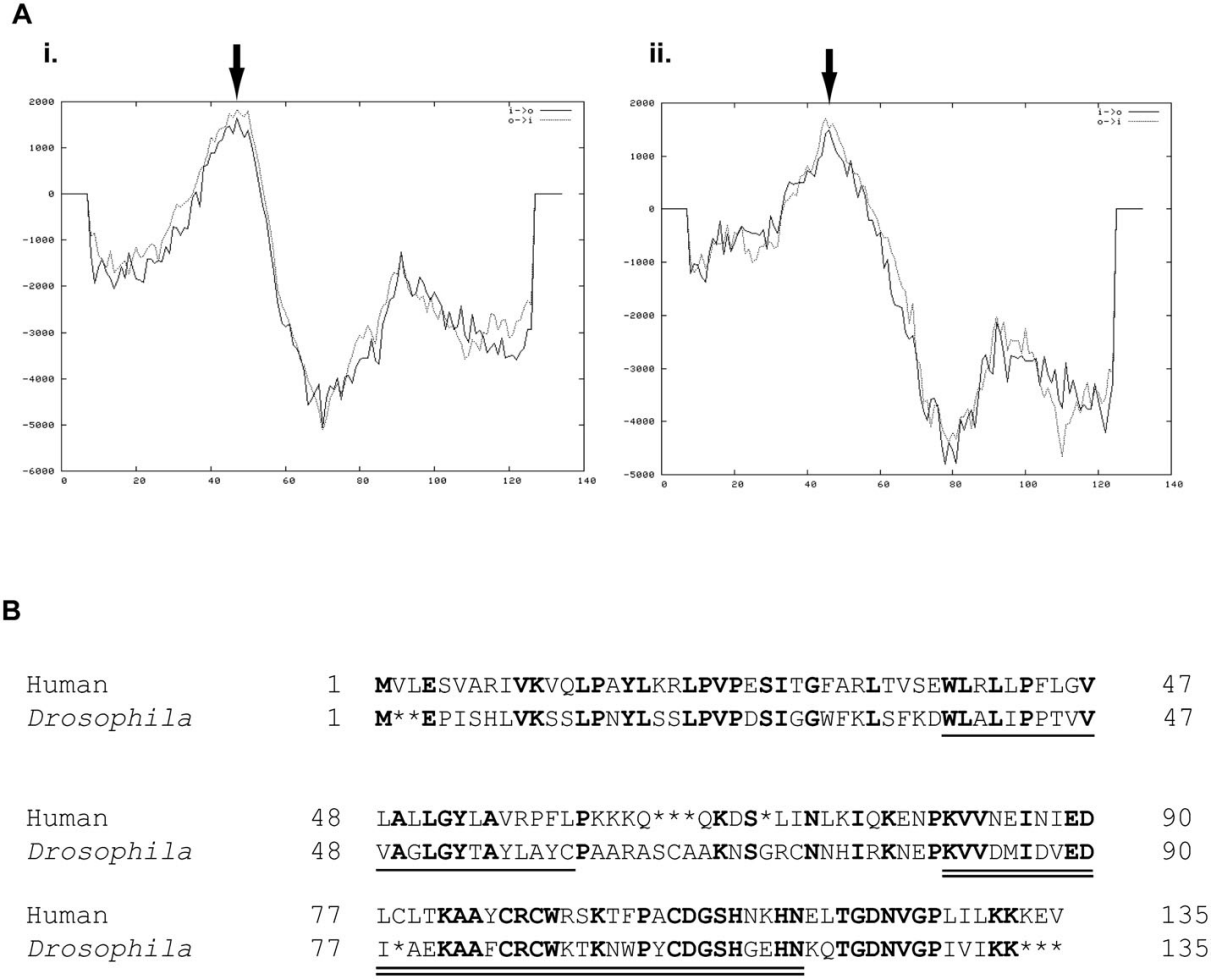
Conserved structure of the *CISD2* and *cisd2* gene products. (A) Hydropathy plots for gene products from human *CISD2* (i) and fly *cisd2* (ii). Amino termini are on the left. Predicted transmembrane domains (TMpred at embnet) are indicated by arrows. (B) Comparison of the primary amino acid sequences for the two gene products. Gaps are represented by asterisks (*). Identical amino acids are in bold. The single underline represents the predicted transmembrane domains. The double underline represents the CDGSH domains.

BLASTp (Altschul et al., 1997) searches of fly annotated proteins with the predicted gene product of human *CISD2* identified *CG1458* as the best orthologue in *Drosophila*. The *CG1458* and *CISD2* predicted proteins are 46% identical and 68% similar in primary amino acid sequence, very similar in size (135 and 133 amino acids, respectively) and have the same predicted topology (Fig. 1A). Additionally, both proteins contain a single predicted transmembrane domain (Fig. 1A) as well as a single predicted CDGSH iron–sulfur domain at the same position (Fig. 1B). BLASTp (Altschul et al., 1997) searches of human annotated proteins with the fly *CG1458* translation product identified *CISD2* as the best human orthologue, although the human *CISD1* locus encodes a protein that also shares considerable homology to that of *CG1458* (31% identical, 48% similar, single predicted transmembrane domain, single CDGSH iron–sulfur domain). Considering these data, and that we found no other predicted fly proteins with significant homology to the *CISD2* gene product, we have designated *CG1458* in *Drosophila* as *cisd2*.

**Fig. 2.**
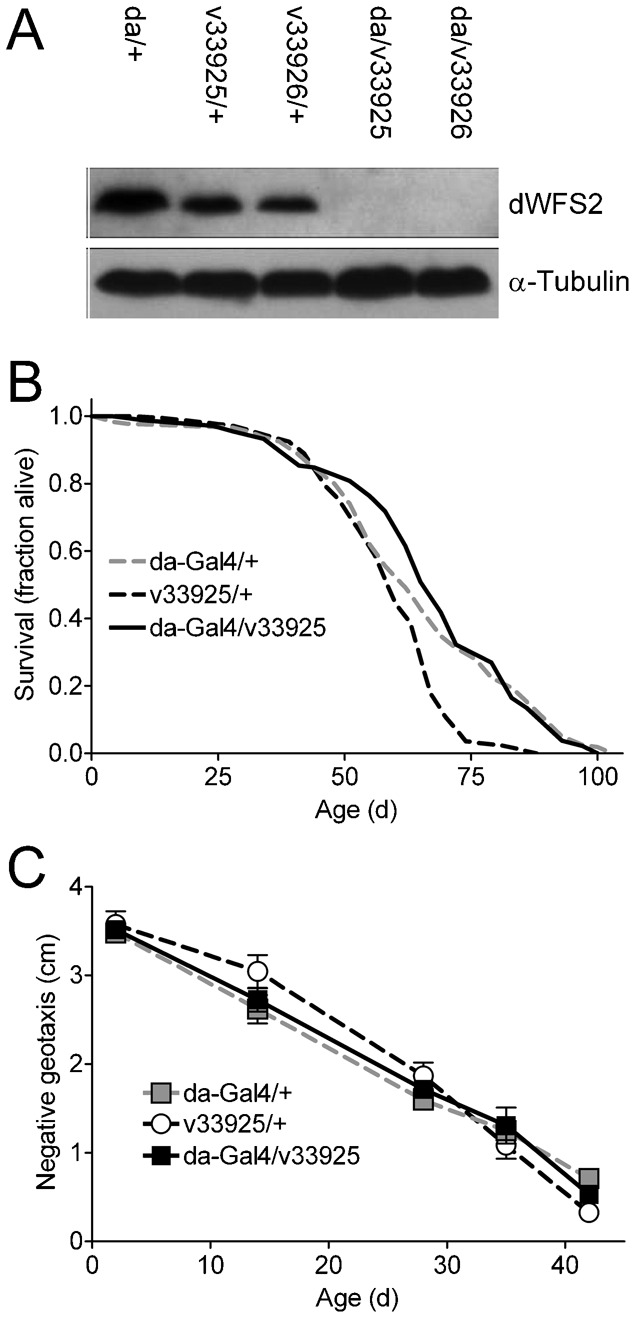
Ubiquitous knockdown of *cisd2* is not associated with obvious detrimental effects. (A) Knockdown of dWFS2 protein. dWFS2 protein was robustly detected with anti-CISD2 antisera in extracts from control animals (*da/+*, *v33925/+ and v33926/+*) whereas it was undetectable in extracts from *cisd2* knockdown flies (*da/v33925 and da/v33926*). Top panel, dWFS2 (∼14.5 kDa); bottom panel, α-tubulin (∼50 kDa) loading control. (B) Survival under normal housing conditions was not altered by ubiquitous expression of *v33925* (*da-Gal4*/*v33925*) compared to controls (*v33925*/+, *da-Gal4*/+) (log-rank test, n.s.). (C) Ubiquitous knockdown of *cisd2* (*da-Gal4*/*v33925*) did not alter locomotor performance (negative geotaxis) across age compared to controls (*da-Gal4*/+, *v33925/+*) (two-way ANOVA, n.s.).

We used the *Gal4-UAS* system (Brand and Perrimon, 1993) to drive two RNA interference (RNAi) transgenes to manipulate *cisd2* expression. *da-Gal4*-driven ubiquitous expression of *UAS-cisd2*-RNAi transgenes *v33925* and *v33926* (Dietzl et al., 2007) decreased *cisd2* mRNA levels by 99±0.1% and 97±0.4%, respectively (*n* = 3) as determined by quantitative real-time PCR (qRT-PCR). The *cisd2* RNAi transgenes do not have predicted off-target effects (see Materials and Methods) and do not alter expression of *Drosophila wfs1* (the fly orthologue of the causative gene for WFS1, data not shown). A protein band consistent with the size of the *cisd2* translation product was readily detectable on immunoblots of extracts from control flies, but not from flies with ubiquitous expression of *cisd2* RNAi transgenes *v33925* or *v33926* (Fig. 2A). These qRT-PCR and immunoblot results indicate that expression of the *cisd2* RNAi transgenes causes a strong loss of function in *cisd2*, although they do not rule out the possibility that some residual expression of *cisd2* remains in these animals.

### Knockdown of *cisd2* alone does not have obvious detrimental effects in *Drosophila*

**Fig. 3.**
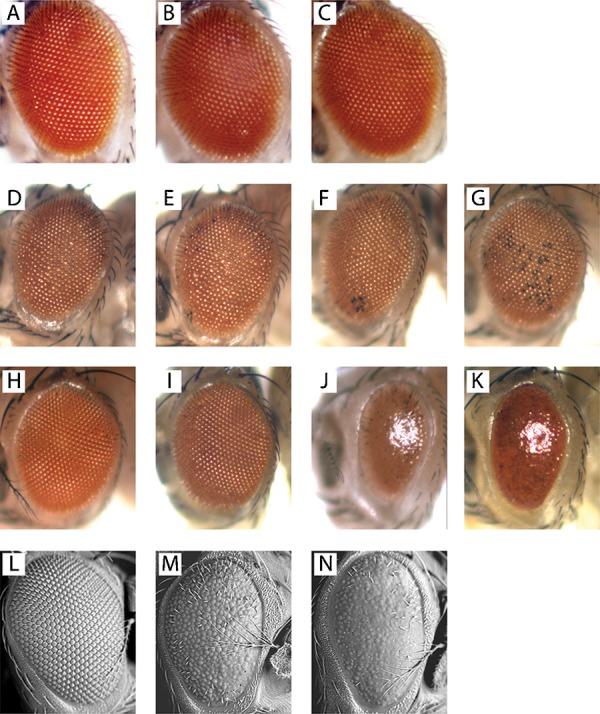
Effect of *cisd2*, *Ppt1* and *cln3* on external eye morphology. Light microscopic images (A–K) or scanning electron micrographs (L–N) of representative genotypes. (A) Normal eye morphology in *gmr-Gal4*/+, (B) *gmr-Gal4*/*v33925* and (C) *gmr-Gal4*/*v33926*. (D–G) Representative *gmr-Gal4*/*UAS-Ppt1* flies with no (D), slight (E), mild (F) or moderate (G) black ommatidia. (H,L) *cisd2^G6528^* mutants with normal eye morphology. (I) Normal eye morphology in *gmr-Gal4*/*UAS-Ppt1* in a *cisd2^G6528^* mutant background. (J,M) *gmr-Gal4*/*UAS-cln3* dysmorphic eye. (K,N) Enhanced eye dysmorphology in *gmr-Gal4*/*UAS-cln3* in a *cisd2^G6528^* mutant background.

As an initial step toward a genetic analysis of *cisd2* in *Drosophila*, we determined whether knocking down its expression in several different tissues led to obvious phenotypes in adults reared and aged under normal housing conditions. Knockdown of *cisd2* throughout the body (*da-Gal4*, Fig. 2B,C; *Actin*-*Gal4*, supplementary material Table S2), in the musculature (*mef2*-*Gal4*, supplementary material Table S2), or in the nervous system (*elav*-*Gal4*, *188Y*-*Gal4* and *Appl*-*Gal4*, supplementary material Table S2) had no obvious effects on lifespan or age-related locomotor impairment. Additionally, knockdown of *cisd2* ubiquitously (*da-Gal4*), in the musculature (*mef2*-*Gal4*) or in the nervous system (*Appl*-*Gal4*) did not lead to a change in bang sensitivity (an index of seizure susceptibility (Fergestad et al., 2006)) in 1–8-week-old flies (supplementary material Table S2). Ubiquitous knockdown of *cisd2* via *da-Gal4* had no significant effect on expression of *4E-BP* at 1 or 8 weeks of age (supplementary material Table S2), suggesting that insulin signaling was not impaired in these animals (Fuss et al., 2006). Expression of *cisd2* RNAi in the eye via *gmr*-*Gal4* (Freeman, 1996) also had no discernible effect on external eye morphology (Fig. 3A–C). Thus, knockdown of *cisd2* via a number of *Gal4* drivers does not appear to have major negative consequences on several independent measures in flies housed under normal laboratory conditions.

To address whether *cisd2* might be important for stress sensitivity in *Drosophila*, we evaluated whether ubiquitous knockdown of *cisd2* altered survival when flies were exposed to thermal, desiccation, starvation, oxidative (hyperoxia, paraquat and H_2_O_2_), FeCl_3_ (iron overload) and tunicamycin (ER) stress. We assessed survival of flies at 1 and 6 weeks of adulthood to address the possibility that effects of *cisd2* knockdown might manifest with age. Although we occasionally saw subtle effects of ubiquitous knockdown of *cisd2* on stress sensitivity in individual experiments, these effects were not consistently observed (supplementary material Table S2). Additionally, expression of *cisd2* RNAi selectively in the nervous system and musculature had no consistent effect on sensitivity to exogenous stressors (supplementary material Table S2). Knockdown of *cisd2*, therefore, had no discernible effect on sensitivity to any of the stressors we tested.

### Targeted genetic analysis identifies a novel interaction between *cisd2* and *Ppt1*

Given that mutations in *CISD2* cause neurodegeneration in WFS2, we postulated that *cisd2* might interact with genes known or predicted to cause other forms of neuropathology in flies. We therefore assessed whether *gmr*-*Gal4*-driven expression of *cisd2* RNAi modified the phenotypes in several genetic models of neurodegeneration (autosomal dominant retinitis pigmentosa, ataxia telangiectasia, Parkinson disease, Alzheimer disease, etc., supplementary material Table S3). Additionally, we determined whether *gmr*-*Gal4*-driven expression of *cisd2* RNAi led to a synthetic phenotype in conjunction with altered cellular processes associated with pathology (oxidative stress, apoptosis and autophagy, supplementary material Table S3).

Light microscopic analyses in a small-scale screen with ∼50 flies/genotype suggested that knockdown of *cisd2* modified the external eye morphology in two strains that overexpressed wild-type *Drosophila palmitoyl-protein thioesterase 1* (*Ppt1*). Knockdown of *cisd2*, however, had no discernible effect in any of the other strains tested (supplementary material Table S3). Overexpression of *Ppt1* in the *Drosophila* eye causes blackened ommatidia thought to be indicative of apoptosis (Korey and MacDonald, 2003). In humans, loss of function mutations in *PPT1* cause infantile neuronal ceroid lipofuscinosis, a severe pediatric neurodegenerative disease resulting in death by 10 years of age (Vesa et al., 1995).

**Fig. 4.**
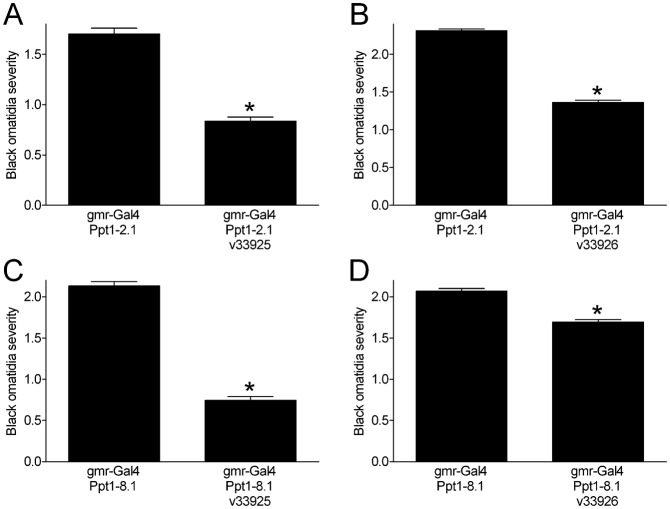
*cisd2* RNAi suppresses the effects of *Ppt1* overexpression in the eye. *gmr-Gal4*-driven expression of two independent *UAS-Ppt1* transgenes (*Ppt1-2.1* and *Ppt1-8.1*) alone or with *cisd2* RNAi caused black ommatidia that varied in severity (representative photographs in Fig. 2). The severity of black ommatidia in each eye was scored on a four-point scale for quantification: none (0), slight (1), mild (2) and moderate (3). (A,B) *gmr-Gal4*/*UAS-PPT1-2.1* alone, with *v33925* or with *v33926*. (C,D) *gmr-Gal4*/*UAS-Ppt1-8.1* alone, with *v33925* or with *v33926*. Expression of *v33925* or *v33926 cisd2* RNAi decreased the severity of *Ppt1*-induced black ommatidia in all cases (*individual Mann–Whitney tests, p<0.0001, *n* = 310–396 per genotype). Data are compiled from two independent experiments.

We pursued the possibility that knockdown of *cisd2* modifies the *Ppt1* overexpression phenotype by performing a series of larger single-blind studies that included more than 300 eyes per genotype. As previously reported (Korey and MacDonald, 2003), we found that overexpression of *Ppt1* in the fly eye via two independent transgenes (*Ppt1-2.1* and *Ppt1-8.1*) led to a blackened ommatidia phenotype with variable expressivity (Fig. 3D–G). We formally quantitated the severity of the black ommatidia phenotype in each eye using a four-point scale: normal (no black ommatidia, 0); slight (a few black ommatidia, 1); mild (one or more small patches of black ommatidia, 2); or moderate (black ommatidia throughout the eye, 3) (Fig. 3D–G). We then compiled the data for genotypes expressing *Ppt1* alone or concurrently with the *cisd2* RNAi transgenes. These larger studies confirmed that *cisd2* knockdown (via the *v33925* RNAi transgene) partially suppressed (i.e. decreased the severity of) the black ommatidia phenotype due to overexpression of two independent *Ppt1* transgenes (Fig. 4A, *Ppt1-2.1*; Fig. 4C, *Ppt1-8.1*). Similarly, knockdown of *cisd2* with the *v33926* RNAi transgene led to a partial suppression of the black ommatidia phenotype in flies overexpressing *Ppt1* (Fig. 4B, *Ppt1-2.1*; Fig. 4D, *Ppt1-8.1*).

**Fig. 5.**
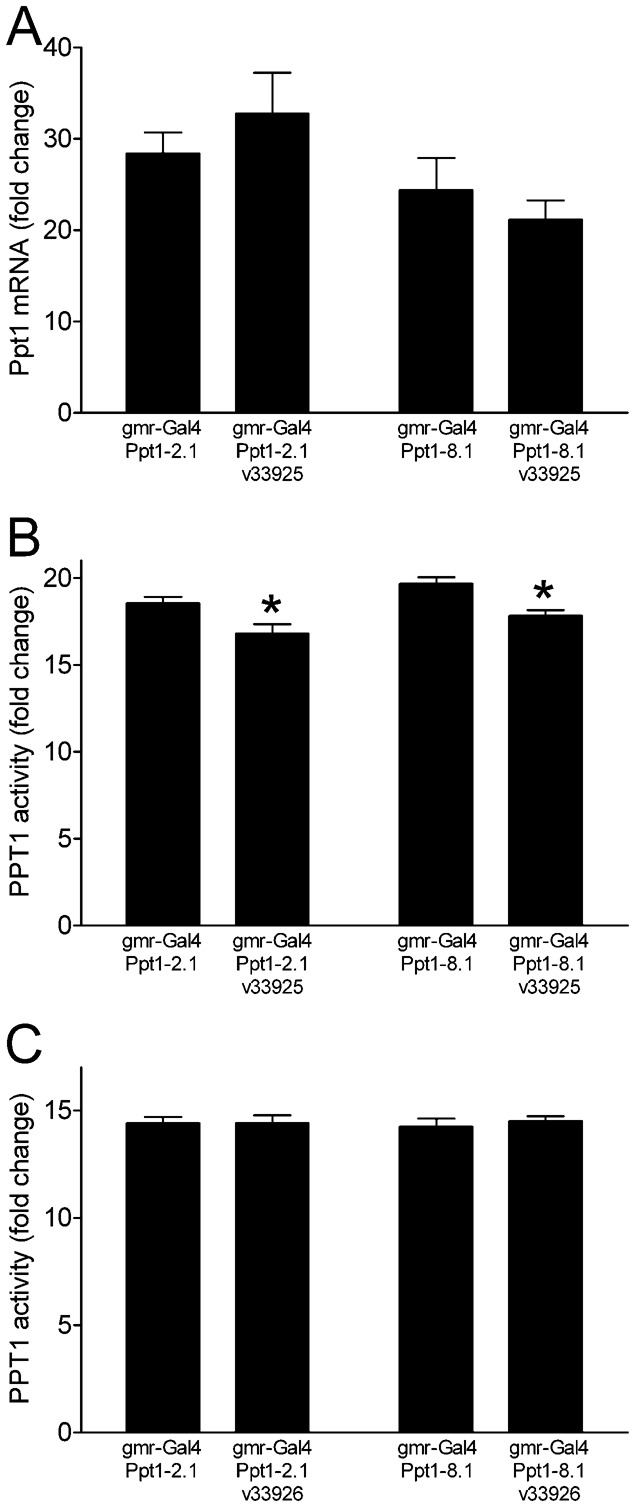
Effect of RNAi-mediated knockdown of *cisd2* on *Ppt1* mRNA expression and enzyme activity. (A) Expression of *v33925* did not alter total head *Ppt1* mRNA expression (individual t tests, n.s., *n* = 4). (B,C) PPT1 enzyme activity in fly heads. (B) Co-expression of the *v33925* RNAi transgene with *Ppt1-2.1* or *Ppt1-8.1* decreased PPT1 enzyme activity (*individual t tests, *n* = 9 per genotype, p = 0.019 and p = 0.002, respectively). (C) Co-expression of the *v33926* RNAi transgene with either *Ppt1-2.1* or *Ppt1-8.1* did not affect PPT1 enzyme activity (individual t tests, *n* = 17–18, n.s.). All data are presented as fold increases relative to endogenous *Ppt1* mRNA or PPT1 activity in heads from *gmr-Gal4*/+ control flies.

To address the possibility that expression of the *cisd2* RNAi transgenes suppressed the severity of black ommatidia simply by blunting the overexpression or function of *Ppt1*, we evaluated *Ppt1* mRNA expression and enzyme activity. We used fly head extracts for these studies because *gmr*-*Gal4* drives expression in the eye (a major portion of the head). Expression of *cisd2* RNAi had no discernible effect on *Ppt1* mRNA overexpression (Fig. 5A). Additionally, although expression of the *v33925* RNAi transgene led to a modest but statistically discernible decrease in PPT1 enzyme activity (Fig. 5B), expression of the v*33926* RNAi transgene did not alter PPT1 enzyme activity (Fig. 5C). The most parsimonious interpretation of our mRNA and enzyme activity studies is that the *cisd2* RNAi-mediated suppression of black ommatidia is unlikely to be due to decreased expression or function of *Ppt1*.

**Fig. 6.**
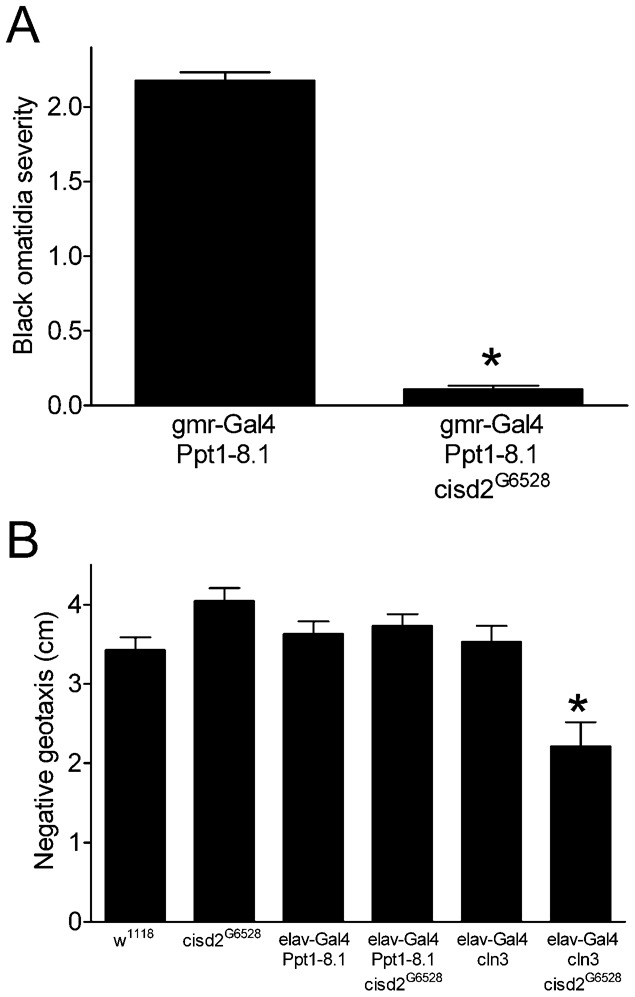
A *cisd2* loss of function mutation interacts with *Ppt1* and *cln3* overexpression. (A) The severity of black ommatidia in flies expressing *Ppt1* was reduced by the *cisd2^G6528^* mutation (*Mann–Whitney test, p<0.0001, *n* = 208–237). (B) Genotype had a significant overall effect on negative geotaxis (one-way ANOVA, p<0.0001, *n* = 6–9). The *cisd2^G6528^* mutation decreased negative geotaxis in flies overexpressing *cln3* in the nervous system (*Bonferroni multiple comparison, p<0.001).

We used a transposon insertion mutation (*P{EP}G6528*) that resides within the protein coding sequence of *cisd2* exon 1 (http://flybase.org) to further address the possibility that *cisd2* influences the effects of *Ppt1* overexpression in the eye. We confirmed the reported location of the *G6528* insertion in exon 1 of *cisd2* using standard PCR on genomic DNA and also found that *G6528* reduced *cisd2* expression to nearly undetectable levels (2.6% of control, one sample t test, p<0.0001, *n* = 3). The insertion site and decreased *cisd2* mRNA expression indicate that *G6528* is a very strong loss of function allele of *cisd2*. Consistent with our RNAi data (Fig. 4), the black ommatidia phenotype from *Ppt1* overexpression was greatly reduced in a *cisd2^G6528^* background (Fig. 3H,I, Fig. 6A). These data confirm that loss of function in *cisd2* modifies the *Ppt1* overexpression eye phenotype.

### *cisd2* exhibits a genetic interaction with *cln3*

Given that *cisd2* is a genetic modifier of *Ppt1*, the fly orthologue of human *PPT1*, we postulated that *cisd2* may interact with additional genes associated with lysosomal storage diseases. We therefore assessed the effect of *cisd2* knockdown on the external eye morphology of flies with altered expression of or mutations in genes associated with several different lysosomal storage diseases (supplementary material Table S4). In initial experiments using light microscopy, knockdown of *cisd2* appeared to enhance the disorganized ommatidia phenotype caused by overexpression of *Drosophila cln3* (data not shown), the orthologue of human *ceroid-lipofuscinosis*, *neuronal 3* (*CLN3*) (Tuxworth et al., 2009). In humans, mutations in *CLN3* cause a juvenile form of neuronal ceroid lipofuscinosis, a lysosomal storage disease. Consistent with our initial RNAi data, the disorganized ommatidia and loss of eye bristles seen with *cln3* overexpression were exacerbated in a *cisd2^G6528^* mutant background as determined by light (Fig. 3H,J,K) and scanning electron microscopy (Fig. 3L–N).

### *cisd2* loss of function and *cln3* gain of function cause a synthetic locomotor phenotype

We addressed the possibility that *cisd2*, *Ppt1* and *cln3* might interact in tissues outside of the eye by assessing negative geotaxis in flies overexpressing *Ppt1* or *cln3* throughout the nervous system in *cisd2* wild-type or mutant backgrounds (Fig. 6B). Negative geotaxis was not altered in *cisd2^G6528^* mutants compared to our standard *w^1118^* laboratory stock, consistent with our previous *cisd2* RNAi studies (Fig. 2; supplementary material Table S2). *elav*-*Gal4*-driven nervous system overexpression of *Ppt1* in a *cisd2* wild-type or mutant background did not affect negative geotaxis, precluding a formal assessment of a possible genetic interaction between these two genes within the context of this behavior. Interestingly, while negative geotaxis was normal in *cisd2^G6528^* mutants and in flies overexpressing *cln3*, flies with concurrent *cisd2* loss of function and *cln3* gain of function had substantial decreases in this behavior (Fig. 6B). This *cisd2/cln3* synthetic phenotype is consistent with our studies showing that *cisd2* loss of function enhances the effect of *cln3* overexpression in the eye (Fig. 3H,J–N).

### Knockdown of *cisd2* does not interact with loss of function in *Ppt1*, *cln3* or related genetic modifiers

Since reduced function of *Ppt1* or *cln3* alone does not cause obvious changes in external eye morphology (Hickey et al., 2006; Tuxworth et al., 2009), we postulated that *cisd2* knockdown in conjunction with *Ppt1* or *cln3* loss of function might lead to a synthetic phenotype in this tissue. Similarly, we postulated that *cisd2* may work in concert with previously identified genes that genetically interact with *Ppt1* and *cln3* (Buff et al., 2007; Tuxworth et al., 2009). We therefore evaluated the external eye morphology in flies harboring loss of function in *Ppt1*, *cln3* or previously reported genetic modifiers of these genes (supplementary material Table S5) alone and with *cisd2* knockdown. Knockdown of *cisd2* in the eye did not lead to obvious changes in the external morphology of the eye in any of these additional studies. These studies suggest that reduced function of *cisd2* does not interact with *Ppt1* or *cln3* loss of function manipulations or genetic modifiers of *Ppt1* or *cln3*. Our studies do not formally rule out these possibilities, however, since our interpretation is based on the lack of a synthetic phenotype.

### A novel interaction between *Ppt1* and *cln3*

**Fig. 7.**
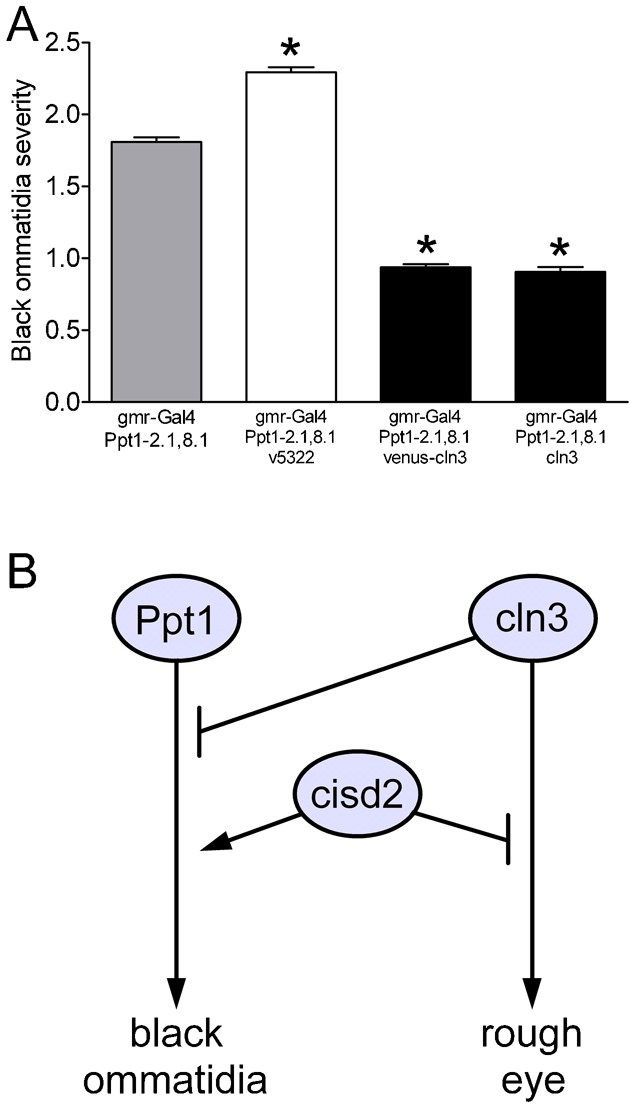
Genetic interaction between *Ppt1* and *cln3* and a proposed genetic model for *cisd2*, *Ppt1* and *cln3* function. (A) The severity of black ommatidia due to *Ppt1* overexpression (grey bar, *gmr-Gal4/Ppt1-2.1,8.1*) was enhanced by *cln3* RNAi (white bar, *gmr-Gal4/Ppt1-2.1,8.1/v5532*) and suppressed by *cln3* overexpression (black bars, *gmr-Gal4/Ppt1-2.1,8.1/venus-cln3* and *gmr-Gal4/Ppt1-2.1,8.1/cln3*) (Kruskal–Wallis ANOVA, p<0.0001; *Dunn's multiple comparison tests, p<0.001; *n* = 178–666/genotype). Data are compiled from two independent experiments. (B) Genetic model for *cisd2*, *Ppt1* and *cln3*. Overexpression of *Ppt1* and *cln3* led to eyes that had black ommatidia or were rough, respectively. *cisd2* is a positive regulator of the *Ppt1* pathway and a negative regulator of the *cln3* pathway. *cln3* is a negative regulator of the *Ppt1* pathway. Arrows in the model could represent the function of multiple genes and are not meant to indicate direct physical interactions between the gene products from *cisd2*, *Ppt1* or *cln3*.

Our studies in flies indicate that *Ppt1* and *cln3* exhibit genetic interactions with *cisd2*, suggesting that *Ppt1* and *cln3* may be functionally linked. To address this possibility, we evaluated external eye morphology in flies expressing several different combinations of *Ppt1* and *cln3* transgenes. We found that *cln3* RNAi (*v5322*) partially enhanced while *cln3* overexpression (*venus-cln3* or *cln3*) partially suppressed the severity of black ommatidia caused by *Ppt1* overexpression (Fig. 7A). Conversely, we found no evidence that *Ppt1* overexpression or loss of function altered the rough eye phenotype due to *cln3* overexpression (not shown). Eyes with concurrent overexpression of both *Ppt1* and *cln3* did exhibit a decrease in pigmentation, but this change could be due to simple additive pathology. These data indicate that *cln3* modifies the *Ppt1* black ommatidia phenotype in *Drosophila* and raise the possibility that *cln3* might normally function as a negative regulator of the *Ppt1* pathway in flies.

### Genetic and gene network models for the coordinated function of *cisd2*, *Ppt1* and *cln3*

Our data support a model in which *cisd2*, *Ppt1* and *cln3* function in concert in flies (Fig. 7B). In this model, endogenous *cisd2* is a positive regulator of the pathway leading from *Ppt1* overexpression to black ommatidia while it is a negative regulator of the pathway leading from *cln3* overexpression to disorganized ommatidia. Additionally, *cln3* antagonizes the *Ppt1* pathway leading to black ommatidia in our model. Importantly, the arrows in our model (Fig. 7B) could represent any number of genes involved in the eye phenotypes caused by overexpression of *Ppt1* or *cln3*. Our studies are the first to support a model for the coordinated function of *cisd2*, *Ppt1* and *cln3* in any species.

To better understand the model in Fig. 7B, we used gene network analyses in GeneMania (Mostafavi et al., 2008; Warde-Farley et al., 2010) to identify known or predicted pair-wise gene interactions for orthologues of *cisd2*, *Ppt1* and *cln3* (i.e. seed genes) in *S. cerevisiae*, *C. elegans*, *Drosophila*, mice and humans. Interactions in GeneMania are defined by gene pairs that are co-expressed, have demonstrated or predicted genetic interactions, or encode gene products that physically interact, have shared protein domains or co-localize (Mostafavi et al., 2008; Warde-Farley et al., 2010).

**Fig. 8.**
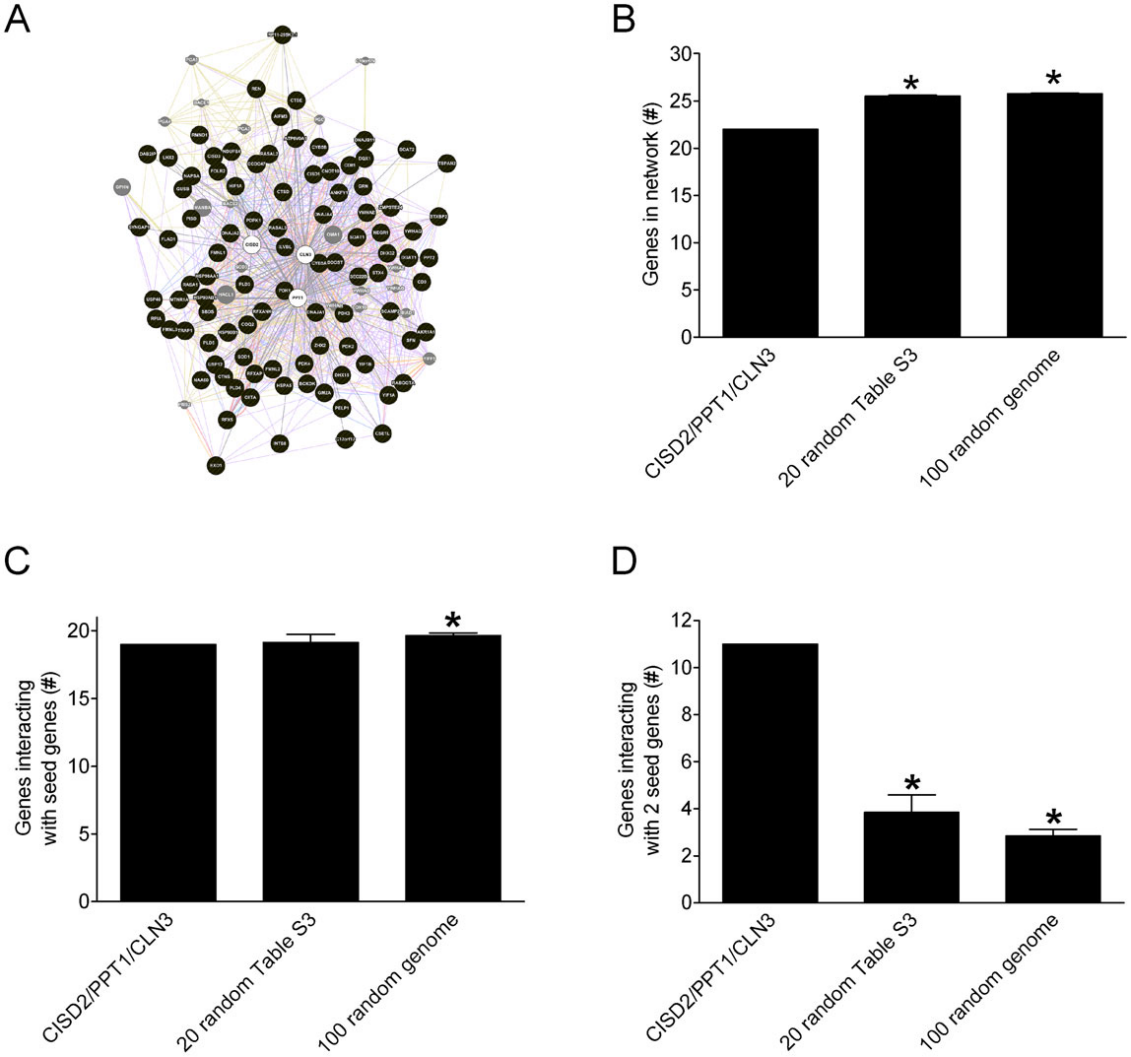
The *CISD2/PPT1/CLN3* multi-species interaction network. (A) GeneMania network depiction using human gene symbols. Seed genes (*CISD2*, *PPT1* and *CLN3*) are indicated by open circles. Filled circles represent genes predicted to interact with seed genes. Smaller grey circles represent genes predicted by GeneMania to also be in the network. Lines represent GeneMania interactions between genes. Total number of genes (B), number of genes that interact with at least one seed gene (C) and the number of genes that interact with two seed genes (D) in the *CISD2/PPT1/CLN3* network, for 20 randomly chosen sets of three seed genes from supplementary material Table S3 and 100 randomly chosen sets of three genes from the human genome. The total number of genes was significantly greater in the randomly seeded networks (panel B, one-sample t test, p<0.0001). The number of network genes that interacted with at least one seed gene was comparable in all networks (panel C, individual Fisher's exact tests, n.s.), but the number of genes that interacted with two seed genes was significantly higher in the *CISD2/PPT1/CLN3* network (*individual Fisher's exact tests; 20 networks from supplementary material Table S3, p = 0.017; 100 networks from human genome, p = 0.0051).

We compiled all genes from GeneMania known or predicted to interact with orthologues of *cisd2*, *Ppt1* and *cln3* in humans, mice, flies, worms and yeast (supplementary material Table S6) and then converted all of the interacting orthologues to human gene symbols for convenience (supplementary material Table S7) (gProfiler; Reimand et al., 2011). The resulting multi-species interaction gene network contains 117 human genes total, with 99 genes known or predicted to interact with *CISD2*, *PPT1* or *CLN3* (the human orthologues of fly *cisd2*, *Ppt1* and *cln3*, respectively) (Fig. 8A). Approximately one-third of the gene–gene interactions in the *CISD2/PPT1/CLN3* multi-species interaction network were based on co-expression data, while the remainder of the interactions was based on other results (supplementary material Table S8). We identified 32 genes (collectively from the five species queried) that interacted with two seed genes used to derive the network (supplementary material Table S9). The *CISD2/PPT1/CLN3* multi-species interaction network as a whole is over-represented for genes involved in carbohydrate metabolism, chaperone/protein folding and endopeptidases/proteases (Huang et al., 2009a; Huang et al., 2009b), suggesting that these processes might underlie disease states associated with altered function of *CISD2*, *PPT1* and *CLN3*.

To address the possibility that the *CISD2/PPT1/CLN3* network – and more specifically the gene–gene interactions that defined it – arose from random chance, we compared gene–gene interactions between *CISD2/PPT1/CLN3* network genes derived from human data (supplementary material Table S6) to interactions within 120 additional GeneMania networks seeded with randomly selected sets of human genes (supplementary material Table S10). The randomly selected seed genes (in sets of 3 to mirror the scope of the seed genes in the *CISD2/PPT1/CLN3* network) were from (i) the genes or orthologues of genes in supplementary material Table S3 that showed no interaction with knockdown of fly *cisd2* and (ii) annotated genes from the human genome as a whole (Meyer et al., 2013). The total number of genes was somewhat higher (Fig. 8B) while the number of genes that interacted with seed genes was comparable (Fig. 8C) in the randomly generated and the *CISD2/PPT1/CLN3* networks. Strikingly, the number of genes that interacted with two of the three seed genes (i.e. bivalent interactors) was substantially higher in the *CISD2/PPT1/CLN3* network compared to the 120 networks seeded with randomly selected genes (Fig. 8D). This analysis indicates that the *CISD2/PPT1/CLN3* network has a more complex, multi-valent structure around the seed genes than would be expected by chance alone. Therefore, this network could be informative regarding the collective function of *CISD2*, *PPT1* and *CLN3*.

Given the severe clinical neuropathology in patients with mutations in *CISD2*, *PPT1* and *CLN3* and the genetic interactions between these genes in flies, we postulated that the *CISD2/PPT1/CLN3* gene network might be broadly involved in human neurological conditions. To examine this gene network specifically in neurodevelopmental disorders, we searched human genetic data for variants within *CISD2*, *PPT1*, *CLN3* and the other 19 human genes identified by network analysis in humans (supplementary material Table S6). Individually rare but collectively common CNVs are known to be enriched in cases with neurodevelopmental disorders including intellectual disability and congenital malformations, autism, schizophrenia, congenital cardiac disease and epilepsy (Girirajan and Eichler, 2010; Girirajan et al., 2011). We compared the frequencies of rare CNVs (deletions and duplications combined) encompassing *CISD2*, *PPT1*, *CLN3* and their gene network partners in unaffected controls and in individuals with intellectual disability phenotypes. Depending on the probe coverage sufficient to make high confidence CNV calls, the total number of cases evaluated ranged from 8,300 to 58,120 (Table 1). We found a small but significant overall enrichment for CNVs for the 22 genes tested in affected individuals (0.35%) versus controls (0.30%). Additionally, we found statistically significant enrichment for CNVs encompassing *CLN3* and *SCAMP2* in affected individuals (Table 1). We note that limited statistical power could have impacted our ability to detect enrichment for rare variants for other genes in the cases compared to controls.

**Table 1.**
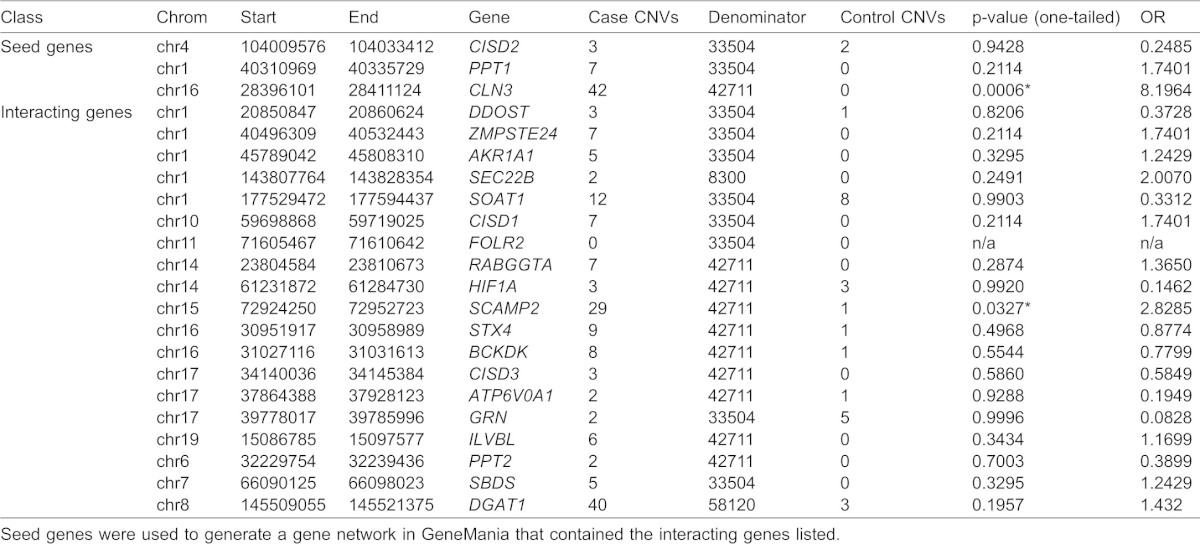
Analysis of CNVs in genes of the CISD2/PPT1/CLN3 network

## DISCUSSION

WFS1 and WFS2 are caused by mutations in *WFS1* (Strom et al., 1998) and *CISD2* (Amr et al., 2007), respectively. Although we are beginning to better understand the biochemical properties of the *CISD2* gene product (Amr et al., 2007), the genes and genetic pathways associated with *CISD2* have not been characterized. Here, we performed a series of genetic and bioinformatic analyses to identify molecular pathways associated *CISD2* function.

We used RNAi and a mutation to determine whether decreased function of *cisd2* (the fly orthologue of *CISD2*) led to obvious phenotypes in *Drosophila*. Surprisingly, flies with strong loss of function in *cisd2* alone appeared remarkably healthy under standard housing conditions and when subjected to various exogenous stressors. While we do not currently understand why flies with *cisd2* knockdown were seemingly unperturbed, several possibilities exist. One possibility is that *cisd2* knockdown could be deleterious only under prescribed environmental conditions such as in the presence of certain microbial pathogens as found in mouse models of cystic fibrosis (Davidson et al., 1995). It is also possible that there is a functionally redundant gene in flies that can compensate for reduced expression of *cisd2*. Although it is difficult to formally exclude this possibility, *cisd2* is the only *CISD2* orthologue in flies, and, importantly, reduced expression of *cisd2* modifies *Ppt1* and *cln3* pathology. Thus, if there is a functionally redundant or compensatory gene in flies, it is not structurally homologous to *cisd2* and it cannot compensate for reduced *cisd2* expression in all experimental conditions.

Toward identifying genes that function in concert with *cisd2*, we determined whether loss of function in *cisd2* modified the eye phenotypes in several previously described models of neurodegeneration in *Drosophila*. We found that RNAi-mediated knockdown and a loss of function mutation in *cisd2* suppresses the black ommatidia phenotype caused by fly *Ppt1* overexpression and that *cisd2* loss of function enhances the disorganized ommatidia phenotype caused by overexpression of fly *cln3*. Through follow-up studies we also found that altered *cln3* expression modifies the severity of black ommatidia caused by *Ppt1* overexpression. Our studies in flies support a novel model in which *cisd2*, *Ppt1* and *cln3* function in concert. Additionally, our gene network analyses suggest that *cisd2*, *Ppt1* and *cln3* (and their orthologues in *S. cerevisiae*, *C. elegans*, mice and humans) might be functionally connected to many other genes, including genes that regulate carbohydrate metabolism, chaperone/protein folding and endopeptidases/proteases.

Our studies found that CNVs encompassing human *CLN3* and *SCAMP2* are associated with neurodevelopmental disorders. *CLN3* maps to chromosome 16p11.2 distal to regions previously associated with developmental delay, autism and obesity (Weiss et al., 2008; Bachmann-Gagescu et al., 2010; Bochukova et al., 2010; Rosenfeld et al., 2010; Walters et al., 2010). Notably, *CLN3* is frequently deleted or duplicated in individuals carrying atypical CNVs involving either the autism or the obesity-associated regions. Recently, Pebrel-Richard and colleagues reported a case with a large heterozygous deletion on chromosome 16p11.2 encompassing *CLN3* and a 1.02 kb deletion on the non-deleted allele of *CLN3*. This individual showed features of juvenile ceroid lipofuscinosis or Batten disease in addition to features of developmental delay, attention deficit disorder, and seizures (Pebrel-Richard et al., 2014). Together, these studies suggest that disruption of *CLN3* or possibly other genes in the *CISD2/PPT1/CLN3* interaction network could play a role in several pathological states.

At this time, we can only speculate about the mechanistic connections between *CISD2*, *PPT1*, *CLN3* and their network genes. One possibility is that PPT1-mediated de-palmitoylation of the gene products for *CISD2*, *CLN3* and other network genes is important for their degradation or subcellular localization and therefore function (Smotrys and Linder, 2004). Another possibility is that *CLN3*-mediated signaling via Notch and JNK or synthesis of sphingolipids (Buff et al., 2007; Persaud-Sawin et al., 2007; Tuxworth et al., 2009) might be important for the function of *CISD2*, *PPT1* or other genes in the network. Yet another possibility is that *CISD2*, *PPT1* and *CLN3* are functionally connected via one or more of the other genes in the network through an as yet unidentified biochemical pathway. Our studies provide the rational framework for further investigating these possibilities and therefore the functional connections between genes in the *CISD2/PPT1/CLN3* network. Such studies could lead to a better understanding of the pathogenesis of WFS, lysosomal storage diseases and neurodevelopmental disorders.

## MATERIALS AND METHODS

### *Drosophila* husbandry, strains, and genetics

Fly husbandry and aging were performed as described (Gargano et al., 2005). *da-Gal4*, *mef2*-*Gal4*, *188Y*-*Gal4*, *appl*-*Gal4*, *cisd2^G6528^* and all *Ppt1* modifiers listed in supplementary material Table S5 were obtained from the Bloomington Drosophila Stock Center (Bloomington, IN). *UAS*-RNAi transgenic lines for *cisd2* (*v33925* and *v33926*), *cln3* (*v5322*) and *cln7* (*v5089* and *v5090*) were purchased from the Vienna Drosophila RNAi Center (Vienna, Austria) (Dietzl et al., 2007). The *cisd2* RNAi transgenes do not have predicted off-target effects (defined as genes with at least one continuous stretch of 19 nucleotides complementary to any region of the RNAi transgene (http://stockcenter.vdrc.at/control/vdrcdefinition)). The *gmr*-*Gal4*-*v33925* and the *gmr*-*Gal4*-*v33926* double transgenic flies were created by recombining the *gmr*-*Gal4* element and the *cisd2* RNAi transgenes onto the same chromosome. *UAS-Ppt1* transgenic strains are previously described (Korey and MacDonald, 2003). *Ppt1* loss of function mutant strains (*Ppt1^A179T^* and *Ppt1^S77F^*) were provided by Robert Glaser (Wadsworth Center, Albany NY). The *cln3* overexpression strain (*UAS-cln3* no. 4) was provided by Richard Tuxworth (Kings College London, London, UK). Sources for all other strains are indicated in supplementary material Tables S3, S4, S5.

### *Drosophila* behavioral assays

Negative geotaxis (startle-induced climbing) was analyzed in Rapid Iterative Negative Geotaxis (RING) assays as described previously (Gargano et al., 2005) with 125 animals per genotype. Lifespan was assessed as previously described (Martin et al., 2009). Bang sensitivity was assessed by determining the climbing latency (i.e. time to recovery) in groups of 25 flies after being vortexed in a vial for 15 seconds at the highest setting using a Diagger Vortex Genie 2 (Fergestad et al., 2006).

### Quantitative real-time PCR

mRNA expression was assessed via quantitative real-time PCR (qRT-PCR) studies as previously described (Jones et al., 2009). Briefly, groups of 25 male flies or ∼800 fly heads were frozen at −80°C. Total RNA was isolated using TRIZOL (Invitrogen) and reverse transcribed using oligo(dT) primers and Superscript II reverse transcriptase (Invitrogen). qRT-PCR was performed using an Applied Biosystems Fast 7500 system with SYBR Green PCR master mix (Quanta Biosciences). All SYBR green assays were performed in triplicate and normalized to *Actin5c* mRNA expression. Each qRT-PCR experiment was repeated three times with three independent RNA isolations and cDNA syntheses. Primer information is listed in supplementary material Table S1.

### *Drosophila* stress tests

All flies for stress tests were collected at 1–3 days of age and were tested for stress sensitivity at 1 and 6 weeks of age. In each experiment, the number of dead flies was recorded for each stress test every 4–8 hrs until all flies were dead. Three vials of 25 flies each were tested for each group. For starvation studies, flies were housed in food vials containing 1% agar. For desiccation studies, flies were housed in empty vials placed in a box with desiccant. To assess thermal stress, flies were placed in vials containing 1% agar with 5% sucrose in a 36°C incubator. Hyperoxia studies were performed by placing flies in standard food vials in an air tight container charged with 95% O_2_ twice daily. All drug tests compared survival in drug-treated and vehicle-treated food vials. For Tunicamycin treatment, flies were placed into vials with food pre-treated with 100 µl 2 mM Tunicamycin in 95% ethanol or 95% ethanol (vehicle). Flies were exposed to paraquat, FeCl_3_, and H_2_O_2_ by placing them in vials with 2 Whatman paper discs treated with 300 µl of 5% sucrose (vehicle) or 5% sucrose supplemented with 40 mM paraquat, 200 mM FeCl_3_, or 30% H_2_O_2_, respectively.

### PPT1 activity assay

PPT1 enzyme activity levels were measured as described previously (Buff et al., 2007). Briefly, a single fly head was placed in a well of a 96 well plate on ice with ∼15 heads used per genotype. Heads were crushed by a pestle in 30 µl solution consisting of 20 µl H_2_O and 10 µl of the PPT1 fluorogenic substrate (4-MU-6S-palm-β-glc) and incubated for 2 hours at 30°C. PPT1 activity was measured by the absorbance change at 460 nm. *Ppt1* loss of function flies were used as a negative control.

### Immunoblots

Protein was isolated from 25 flies per genotype by homogenization in radioimmunoprecipitation RIPA lysis buffer containing protease inhibitor cocktail (Roche 1:25 dilution in lysis buffer). Samples were sonicated, incubated on ice for 45 minutes and centrifuged at 16,000 × g for 15 minutes at 4°C. Supernatants were transferred to a new tube and protein concentration was measured using the DC Protein Assay (Bio-Rad). Protein extracts were electrophoresed via SDS-PAGE and transferred to polyvinylidene difluoride (PVDF) membranes. Western blots were probed with a rabbit anti-mouse polyclonal antibody against the *CISD2* gene product (ProteinTech, 1:1,000 dilution in 5% BSA in Tris-buffered saline solution containing 0.1% Tween-20 (TBST) to detect *Drosophila* WFS2 (dWFS2) or a mouse anti-α tubulin monoclonal antibody (Sigma, 1:1,000 dilution in 5% milk in TBST) to detect the loading control. Expression of dWFS2 and α-tubulin was visualized with goat anti-rabbit IgG-HRP (BioRad, 1:10,000 dilution in 5% milk in TBST) and goat anti-mouse IgG-HRP (Santa Cruz, 1:10,000 dilution in 5% milk in TBST), respectively, in conjunction with Western Lightning chemiluminescence reagent plus (PerkinElmer). Western blot experiments were repeated three times with independent protein extracts.

### Light and electron microscopy

Samples for and images of external eye morphology were processed as previously described (Warrick et al., 1999; Chan et al., 2002).

### Gene network analyses

Gene networks were constructed using GeneMania (Mostafavi et al., 2008; Warde-Farley et al., 2010) with default settings. Interactors from GeneMania, including genes that interacted with more than one seed gene, were identified using Excel (Microsoft, Redmond, WA). Identification of orthologues in *S. cerevisiae*, *C. elegans*, *Drosophila*, mice and humans was performed using g:Profiler (Reimand et al., 2011) and BLASTp (Altschul et al., 1997). Random sets of 3 genes were selected from supplementary material Table S3 and the human genome by sorting the relevant gene list based on a randomly assigned number in Excel. Gene ontology analysis was performed with DAVID (Huang et al., 2009a; Huang et al., 2009b).

### Human disease-associated variation

To examine *CLN3*, *PPT1*, *CISD2* and a set of 19 of their interacting partners in the context of a broader neurodevelopmental phenotype, we evaluated human disease-associated variation data from exome sequencing and copy number variation analysis. Specifically, disruptive *de novo* single nucleotide mutations within the 22 genes were queried in the exome sequencing data from 151 families with severe intellectual disability (de Ligt et al., 2012; Rauch et al., 2012) and 927 families with sporadic autism (O'Roak et al., 2011; Iossifov et al., 2012; Neale et al., 2012; O'Roak et al., 2012; Sanders et al., 2012). We also analyzed CNV data from a clinical laboratory database consisting of 58,120 individuals referred primarily for intellectual disability, developmental delay, and other congenital malformations for deletions and duplications within the genes of interest. These samples from affected individuals were sent to Signature Genomic Laboratories from 2004 through 2013 by geneticists, pediatricians, and neurologists from more than 50 referral centers primarily throughout the United States. The ages of the ascertained cases ranged between 2 to 22 years. Based on self-reported ethnicity, about 75% are of European descent, 7% African or African–American, and 18% belonged to other or mixed ancestry (Cooper et al., 2011). These samples were evaluated by array comparative genomic hybridization (array CGH) experiments with a targeted whole genome bacterial-artificial-chromosome microarray (SignatureChip) or an oligonucleotide-based microarray (Signature-ChipOS, custom-designed by Signature Genomic Laboratories and manufactured by Agilent Technologies or Roche NimbleGen). Microarray hybridizations were performed as described previously (Bejjani et al., 2005; Ballif et al., 2008a; Ballif et al., 2008b; Duker et al., 2010). Control CNV data were curated from single nucleotide polymorphism arrays from 8329 individuals with no overt neurological disorders as described previously (Cooper et al., 2011).

We only included those CNVs in the affected individuals that are rare (<0.1% frequency in controls), large (>300 kb), <50% overlapped with large genomic repeats called segmental duplications, and mapped to putative genes described in this study. Further, we only considered interstitial heterozygous deletions and duplications. Large chromosomal abnormalities such as trisomies and monosomies were excluded from the analysis. We considered all CNVs that overlapped by at least 1 bp with the putative gene of interest and compared frequency of events hitting the genes of interest between cases and controls. Depending on the probe coverage of the genes evaluated, the total number of cases available for analysis ranged from 8,300 to 58,120 individuals (Table 1).

### Experimental subjects

The CNV data were curated from a database in Signature Genomic Laboratories. CNV data from de-identified samples were analyzed for variants in specific genes of interest. All experiments with human data conform to the relevant regulatory standards.

### Statistics

JMP 5.01a (SAS Institute, Cary, NC) was used to analyze lifespan and stress survival data (log-rank tests) and negative geotaxis across age (two-way ANOVA). The severity of black ommatidia (categorical data) was analyzed with nonparametric Mann–Whitney tests or Kruskal–Wallis ANOVA followed by Dunn's multiple comparison using Prism 4.03 (GraphPad Software, San Diego, CA). Data for *Ppt1* mRNA, PPT1 enzyme activity and negative geotaxis at a single age were analyzed with parametric t tests or one-way ANOVAs followed by Bonferroni multiple comparison tests using Prism 4.03. The number and percentage of GeneMania interactors from randomly seeded networks were compared to the *CISD2/PPT1/CLN3* network by two-sided one-sample t tests. Statistical analyses on human CNVs were performed with the hypothesis that rare CNVs encompassing genes of interest would be enriched in cases compared to controls and thus one-tailed Fisher's exact tests were used.

### Resource sharing

Enquiries for reagents described in this article should be directed to the corresponding author (M.G.).

## Supplementary Material

Supplementary Material
