# Supplementary material for: Genetic studies in Drosophila and humans support a model for the concerted function of CISD2, PPT1 and CLN3 in disease
Source: Biol Open. 2014 Apr 4;3(5):342–52. doi: 10.1242/bio.20147559 (PMC4021356; doi:10.1242/bio.20147559)
Supplement: Supplementary Material [file supp_3_5_342__index.html]

Genetic studies in Drosophila and humans support a model for the concerted function of CISD2, PPT1 and CLN3 in disease — Supplementary Material 

# Genetic studies in *Drosophila* and humans support a model for the concerted function of *CISD2*, *PPT1* and *CLN3* in disease

## bio.20147559 Supplementary Material

**Files in this Data Supplement:**

- Table S1 - PCR primers for qRT-PCR analyses of *Drosophila* genes with SYBR Green detection.
- Table S2 - Expression of the *cisd2 v33925* RNAi transgene ubiquitously (*da*-Gal4, *Actin*-Gal4), in the muscle (*mef2*-Gal4) or in the nervous system (*elav*-Gal4, *188Y*-Gal4, *Appl*-Gal4) had no consistent effect on behavior, lifespan, stress sensitivity, or external eye morphology.
- Table S3 - Candidate interactors for *cisd2*.
- Table S4 - Lysosomal storage disease candidate interactors.
- Table S5 - Modifiers of PPT1 overexpression and CLN3 overexpression.
- Table S6 - All MSpIn interactors - all species.
- Table S7 - MSpIn all network genes - human.
- Table S8 - Summary of pair-wise gene-gene interactions in the CISD2/PPT1/CLN3 multi-species interaction network.
- Table S9 - Summary of genes from GeneMania that interact with at least two seed genes in the CISD2/PPT1/CLN3 multi-species network.
- Table S10 - Random sets of genes.
