# Supplementary material for: Genetic studies in Drosophila and humans support a model for the concerted function of CISD2, PPT1 and CLN3 in disease
Source: Biol Open. 2014 Apr 4;3(5):342–52. doi: 10.1242/bio.20147559 (PMC4021356; doi:10.1242/bio.20147559)
Supplement: Supplementary Material [file supp_bio.20147559_Jones_Table_S1.doc]

Table S1. PCR primers for qRT-PCR analyses of *Drosophila* genes with SYBR Green detection.

| **Gene** | **Forward primer** | **Reverse Primer** |
| --- | --- | --- |
| *cisd2* | 5’-GTTGTTGCAGCGTCCGCTG-3’ | 5’-CGGTTCCCGACAGCATCG-3’ |
| *Ppt1* | 5’-GTTCTCCATGATCGGGTCG-3’ | 5’-GAGGAACCTTATCACACTCG-3’ |
| *cln3* | 5’-GTACTGCGAGTCCTTGTCC-3’ | 5’-GGAAGTCCTGATCAGCGATG-3’ |
| *4E-BP* | 5’-CATGCAGCAACTGCCAAATC-3’ | 5’-CCGAGAGAACAAACAAGGTGG-3’ |
| *Actin-5C* | 5’-AGCGCGGTTACTCTTTCACCAC-3’ | 5’-GTGGCCATCTCCTGCTCAAAGT-3’ |
