# Supplementary material for: Genetic studies in Drosophila and humans support a model for the concerted function of CISD2, PPT1 and CLN3 in disease
Source: Biol Open. 2014 Apr 4;3(5):342–52. doi: 10.1242/bio.20147559 (PMC4021356; doi:10.1242/bio.20147559)
Supplement: Supplementary Material [file supp_bio.20147559_Jones_Table_S2.doc]

Table S2. Expression of the *cisd2 v33925* RNAi transgene ubiquitously (*da*-Gal4, *Actin*-Gal4), in the muscle (*mef2*-Gal4) or in the nervous system (*elav*-Gal4, *188Y*-Gal4, *Appl*-Gal4) had no consistent effect on behavior, lifespan, stress sensitivity, or external eye morphology.

| **Experiment** | ***Gal4* Driver** | **Ages Tested** |
| --- | --- | --- |
| **Behavior** |  |  |
| Negative Geotaxis | *da, Actin, mef2, elav, 188Y, Appl* | Week 1, 3, 5, and 7 |
| Bang Sensitivity (seizures) | *da, mef2, Appl* | Weeks 1-8 |
|  |  |  |
| **Longevity** |  |  |
| Lifespan | *da, Actin, mef2, 188Y, Appl* |  |
|  |  |  |
| **Insulin Regulation** |  |  |
| *4E-BP* expression analysis | *da* | Weeks 1 and 8 |
|  |  |  |
| **Stress Sensitivity** |  |  |
| Thermal | *da* | Weeks 1 and 6 |
| Desiccation | *da* | Weeks 1 and 6 |
| Starvation | *da* | Weeks 1 and 6 |
| Hyperoxia (oxidative stress) | *da, Appl* | Weeks 1 and 6 |
| Paraquat (oxidative stress) | *da* | Weeks 1 and 6 |
| H_2_O_2_ (oxidative stress) | *da, Appl* | Week 1 |
| FeCl_3_ (iron overload) | *da, Appl* | Week 1 |
| Tunicamycin treatment (ER stress) | *da, Actin, mef2, 188Y, Appl* | Weeks 1 and 6 |
|  |  |  |
| **Development*/*Degeneration** |  |  |
| External Eye Morphology | *gmr* | Weeks 1 and 6 |
