# Supplementary material for: Genetic studies in Drosophila and humans support a model for the concerted function of CISD2, PPT1 and CLN3 in disease
Source: Biol Open. 2014 Apr 4;3(5):342–52. doi: 10.1242/bio.20147559 (PMC4021356; doi:10.1242/bio.20147559)
Supplement: Supplementary Material [file supp_bio.20147559_Jones_Table_S3.doc]

Table S3. Candidate interactors for *cisd2***.** Candidates (*UAS* transgenes or mutants, column 1) associated with several different human diseases or pathological processes (column 3) were crossed to flies expressing *cisd2* RNAi in the eye (*GMR-v33925*). The effects of *cisd2* RNAi on the external eye morphology associated with expression of the transgenes in column 1 were assessed at 1 and 6 weeks of age (see main text).

| **Candidate** | **Class** | **Disease or Process** | **Source** | **Citation** |
| --- | --- | --- | --- | --- |
| *UAS-Ppt1-2.1* | ectopic expression | Infantile neuronal ceroid lipofuscinosis (INCL) | Christopher Korey, Charleston, SC | (Korey et al., 2003) |
|  |  |  |  |  |
| *UAS-Ppt1-8.1* | ectopic expression | Infantile neuronal ceroid lipofuscinosis (INCL) | Christopher Korey, Charleston, SC | (Korey et al., 2003) |
|  |  |  |  |  |
| *ninaE RH27* | mutant | Autosomal dominant retinitis pigmentosa | Hermann Steller, New York, NY | (Ryoo et al., 2007) |
|  |  |  |  |  |
| *ninaE G69D* | mutant | Autosomal dominant retinitis pigmentosa | Hermann Steller, New York, NY | (Ryoo et al., 2007) |
|  |  |  |  |  |
| *UAS-pATM^T4^-RNAi* | knockdown | Ataxia telangiectasia | Stacey Rimkus, Madison, WI | (Rimkus et al., 2008) |
|  |  |  |  |  |
| *UAS-α-synuclein* | ectopic expression | Parkinson disease | Mel Feany, Boston, MA | (Feany et al., 2000) |
|  |  |  |  |  |
| *UAS-α-synuclein-A30P* | ectopic expression | Parkinson disease | Mel Feany, Boston, MA | (Feany et al., 2000) |
|  |  |  |  |  |
| *UAS-α-synuclein-A53T* | ectopic expression | Parkinson disease | Mel Feany, Boston, MA | (Feany et al., 2000) |
|  |  |  |  |  |
| *UAS-DJ-1α-RNAi* | knockdown | Parkinson disease | Bingwei Lu, Palo Alto, CA | (Yang et al., 2005) |
|  |  |  |  |  |
| *UAS-Pink1-RNAi* | knockdown | Parkinson disease | Rolf Bodmer, La Jolla, CA | (Wang et al., 2006) |
|  |  |  |  |  |
| *UAS-GCC90-EGFP* | ectopic expression | Fragile X associated tremor and ataxia syndrome | Juan Botas, Houston, TX | (Jin et al., 2003) |
|  |  |  |  |  |
| *UAS-GCC90-14J* | ectopic expression | Fragile X associated tremor and ataxia syndrome | Juan Botas, Houston, TX | (Jin et al., 2003) |
|  |  |  |  |  |
| *UAS-GCC90-20J* | ectopic expression | Fragile X associated tremor and ataxia syndrome | Juan Botas, Houston, TX | (Jin et al., 2003) |
|  |  |  |  |  |
| *UAS-dUBQN* | ectopic expression | Alzheimer disease | Ming Guo, Los Angeles, CA | (Ganguly et al., 2008) |
|  |  |  |  |  |
| *UAS-UBQLN1* | ectopic expression | Alzheimer disease | Ming Guo, Los Angeles CA | (Ganguly et al., 2008) |
|  |  |  |  |  |
| *UAS-UBQ-8i* | ectopic expression | Alzheimer disease | Ming Guo, Los Angeles, CA | (Ganguly et al., 2008) |
|  |  |  |  |  |
| *UAS-ATX3-Q27* | ectopic expression | Spinocerebellar ataxia 3 | John Warrick, Richmond VA | (Warrick et al., 2005) |
|  |  |  |  |  |
| *UAS-ATX3-trQ78 (c37.3)* | ectopic expression | Spinocerebellar ataxia | John Warrick, Richmond, VA | (Warrick et al., 2005) |
|  |  |  |  |  |
| *UAS-ATX3-trQ78 (c211.2)* | ectopic expression | Spinocerebellar ataxia | John Warrick, Richmond, VA | (Warrick et al., 2005) |
|  |  |  |  |  |
| *UAS-DJ-1β-RNAi v17214* | knockdown | Parkinson disease | Vienna *Drosophila* RNAi Center | (Dietzl et al., 2007) |
|  |  |  |  |  |
| *UAS-DJ-1β-RNAi v17215* | knockdown | Parkinson disease | Vienna *Drosophila* RNAi Center | (Dietzl et al., 2007) |
|  |  |  |  |  |
| *UAS-DJ-1β* | ectopic expression | Parkinson disease | Nancy Bonini, Philadelphia, PA | (Meulener et al., 2005) |
|  |  |  |  |  |
| *UAS-Pink1 L5* | ectopic expression | Parkinson disease | Ming Guo, Los Angeles, CA | (Clark et al., 2006) |
|  |  |  |  |  |
| *UAS-Pink1 L6* | ectopic expression | Parkinson disease | Ming Guo, Los Angeles, CA | (Clark et al., 2006) |
|  |  |  |  |  |
| *UAS-Parkin-RNAi v47636* | knockdown | Parkinson disease | Vienna *Drosophila* RNAi Center | (Dietzl et al., 2007) |
|  |  |  |  |  |
| *UAS-Parkin C2* | ectopic expression | Parkinson disease | Leo Pallanck, Seattle, WA | (Greene et al., 2003) |
|  |  |  |  |  |
| *UAS-Sod1* | ectopic expression | Oxidative damage | Gabriel Boulianne, Ontario, Canada | (Parkes et al., 1998) |
|  |  |  |  |  |
| *UAS-Sod2-RNAi IR24* | knockdown | Oxidative damage | Vienna *Drosophila* RNAi Center | (Dietzl et al., 2007) |
|  |  |  |  |  |
| *UAS-Sod1-RNAi v31552* | knockdown | Oxidative damage | Vienna *Drosophila* RNAi Center | (Dietzl et al., 2007) |
|  |  |  |  |  |
| *Atg1* | mutant | Autophagy | Stock Collection, Bloomington, IN |  |
|  |  |  |  |  |
| *Atg7* | mutant | Autophagy | Stock Collection, Bloomington, IN |  |
|  |  |  |  |  |
| *UAS-Diap (2)* | ectopic expression | Apoptosis | Stock Collection, Bloomington, IN |  |
|  |  |  |  |  |
| *UAS-Diap (3)* | ectopic expression | Apoptosis | Stock Collection, Harvard, MA |  |
|  |  |  |  |  |
| *H99* | mutant | Apoptosis | Herman Steller, New York, NY | (White et al., 1994) |
|  |  |  |  |  |
| *Xr38* | mutant | Apoptosis | Herman Steller, New York, NY | (Peterson et al., 2002) |
|  |  |  |  |  |
| *hidosmR8P* | mutant | Apoptosis | Herman Steller, New York, NY | (Grether et al., 1995) |
