# Supplementary material for: Genetic studies in Drosophila and humans support a model for the concerted function of CISD2, PPT1 and CLN3 in disease
Source: Biol Open. 2014 Apr 4;3(5):342–52. doi: 10.1242/bio.20147559 (PMC4021356; doi:10.1242/bio.20147559)
Supplement: Supplementary Material [file supp_bio.20147559_Jones_Table_S4.doc]

Table S4. Lysosomal storage disease candidate interactors. Each lysosomal storage disease strain (column 2) was crossed to flies expressing *cisd2 v33925* RNAi in the eye via *gmr-Gal4*.

| **Gene** | **Allele or Manipulation** | **Class** | **Source** | **Citation** |
| --- | --- | --- | --- | --- |
| *Benchwarmer* | *P* | mutant | Karen Schulze, Houston, TX | (Dermaut et al., 2005) |
|  | *P29* | mutant | Karen Schulze, Houston, TX | (Dermaut et al., 2005) |
|  | *E14.1* | mutant | Karen Schulze, Houston, TX | (Dermaut et al., 2005) |
|  | *∆31* | mutant | Karen Schulze, Houston, TX | (Dermaut et al., 2005) |
|  | *∆86* | mutant | Karen Schulze, Houston, TX | (Dermaut et al., 2005) |
|  |  |  |  |  |
| *CG8596* (*CLN7*) | *KG05284* | P-element insertion | Stock Center, Bloomington, IN |  |
|  | *UAS-CG8596-RNAi (v5089)* | knockdown | Vienna *Drosophila* RNAi Center | (Dietzl et al., 2007) |
|  | *UAS-CG8596-RNAi (v5090)* | knockdown | Vienna *Drosophila* RNAi Center | (Dietzl et al., 2007) |
|  |  |  |  |  |
| *cln3* | *UAS-cln3-RNAi (v5322)* | knockdown | Vienna *Drosophila* RNAi Center | (Dietzl et al., 2007) |
|  | *UAS-cln3* | ectopic expression | Richard Tuxworth, London, UK | (Tuxworth et al., 2009) |
