# Supplementary material for: Genetic studies in Drosophila and humans support a model for the concerted function of CISD2, PPT1 and CLN3 in disease
Source: Biol Open. 2014 Apr 4;3(5):342–52. doi: 10.1242/bio.20147559 (PMC4021356; doi:10.1242/bio.20147559)
Supplement: Supplementary Material [file supp_bio.20147559_Jones_Table_S5.doc]

Table S5. Modifiers of *PPT1* overexpression and *CLN3* overexpression. *PPT1* modifiers were obtained from the *Drosophila* Stock Center (Bloomington, IN). *CLN3* modifiers were provided by Jennifer Treisman (Skirball Institute, NYU Medical Institute).

| **Modifier for** | **Gene** | **Allele** |
| --- | --- | --- |
| *Ppt1* overexpression | *Blue cheese* | *EY02503* |
|  | *ubcE2H* | *EP1303* |
|  | *Fasciclin 2* | *EP1462* |
|  | *Fat facets* | *EY02018* |
|  | *Miniature* | *EP406* |
|  | *Kayak* | *EY00283* |
|  | *Saxophone* | *EY04377* |
|  | *Fs(1)N* | *EP1336* |
|  | *CG7023* | *EY00249* |
|  | *CG5991* | *EY03559* |
|  | *CG32138* | *EY03931* |
|  | *CG18177* | *EP3301* |
|  | *CG5859* | *EP2090* |
|  |  |  |
| *cln3* overexpression | *Mago nashi* | *69B* |
|  | *Tsunagi* | *∆18* |
