# Supplementary material for: Genetic studies in Drosophila and humans support a model for the concerted function of CISD2, PPT1 and CLN3 in disease
Source: Biol Open. 2014 Apr 4;3(5):342–52. doi: 10.1242/bio.20147559 (PMC4021356; doi:10.1242/bio.20147559)
Supplement: Supplementary Material [file supp_bio.20147559_Jones_Table_S8.doc]

Table S8. Summary of pair-wise gene-gene interactions in the *CISD2/PPT1/CLN3* multi-species interaction network.

|  | ***S. cerevisiae*** | ***C. elegans*** | ***Drosophila*** | **mouse** | **human** | **all** |
| --- | --- | --- | --- | --- | --- | --- |
| *CISD2* | no orthologue | 6 | 18 | 7 | 4 | 35 |
| *PPT1* | 22 | 1 | 19 | 17 | 12 | 71 |
| *CLN3* | 36 | 49 | 6 | 9 | 20 | 120 |
|  |  |  |  |  |  |  |
| co-expression | 12 | 20 | 18 | 9 | 22 | 81 |
| predicted | 2 | 26 | 4 | 11 | 4 | 47 |
| physical | 18 | 0 | 11 | 0 | 3 | 32 |
| shared protein domain | 1 | 7 | 1 | 6 | 3 | 18 |
| other | 10 | 3 | 0 | 5 | 0 | 18 |
| genetic | 10 | 0 | 6 | 0 | 0 | 16 |
| co-localization | 5 | 0 | 3 | 2 | 3 | 13 |
| pathway | 0 | 0 | 0 | 0 | 1 | 1 |
