# Supplementary material for: Genetic studies in Drosophila and humans support a model for the concerted function of CISD2, PPT1 and CLN3 in disease
Source: Biol Open. 2014 Apr 4;3(5):342–52. doi: 10.1242/bio.20147559 (PMC4021356; doi:10.1242/bio.20147559)
Supplement: Supplementary Material [file supp_bio.20147559_Jones_Table_S9.doc]

Table S9. Summary of genes from GeneMania that interact with at least two seed genes in the *CISD2/PPT1/CLN3* multi-species network.

| **Gene** | **Species** | ***CISD2***  **interaction** | ***PPT1***  **interaction** | ***CLN3***  **interaction** | **Citations** |
| --- | --- | --- | --- | --- | --- |
| *CDC37* | *S. cerevisiae* |  | co-expression  co-localization  physical | genetic | Costanzo-Boone-2010-negative-interactions-intermediate, Roberts-Friend-2000 Huh-O'Shea-2003 BIOGRID-SMALL-SCALE-STUDIES; IREF-GRID; IREF-SMALL-SCALE-STUDIES |
| *CLN2* | *S. cerevisiae* |  | co-expression | genetic  physical | Medintz-Thach-2007; Simmons Kovacs-Haase-2012; Urban-Loewith-2007 Lee-Marcotte-2007 Co-citation, Matia-González-Rodríguez-Gabriel-2011 |
| *COQ2* | *S. cerevisiae* |  | other | co-expression | Gasch-Brown-2001; Orlando-Haase-2008; Lee-Marcotte-2007 Co-citation |
| *HSC82* | *S. cerevisiae* |  | co-localization  genetic  physical | physical | IREF-GRID; Costanzo-Boone-2010-profile-similarity Huh-O'Shea-2003 Gavin-Superti-Furga-2002 A; IREF-BIND-TRANSLATION; IREF-GRID; Krogan-Greenblatt-2006 B; Lee-Marcotte-2007 Protein complexes |
| *HSP82* | *S. cerevisiae* |  | co-localization  physical | physical | Huh-O'Shea-2003 BIOGRID-SMALL-SCALE-STUDIES; Gavin-Superti-Furga-2002 A; IREF-BIND-TRANSLATION; IREF-GRID; Krogan-Greenblatt-2006 B; Lee-Marcotte-2007 Protein complexes; Lee-Marcotte-2007 Protein interactions |
| *MCM16* | *S. cerevisiae* |  | physical | genetic | Costanzo-Boone-2010-profile-similarity; BIOGRID-SMALL-SCALE-STUDIES; IREF-GRID; Wong-Hazbun-2007 |
| *PRP43* | *S. cerevisiae* |  | co-expression | physical | Breitkreutz-Tyers-2010 B; IREF-GRID; Gasch-Brown-2000; Gasch-Brown-2001; Hochwagen-Amon-2005; Hughes-Friend-2000; Spellman-Futcher-1998; Urban-Loewith-2007 |
| *SBA1* | *S. cerevisiae* |  | physical | co-expression | Roberts-Friend-2000; BIOGRID-SMALL-SCALE STUDIES; IREF-GRID; IREF-SMALL-SCALE-STUDIES |
| *WHI3* | *S. cerevisiae* |  | co-expression  co-localization | co-expression  genetic  physical | Gasch-Brown-2001; Knijnenburg-Wessels-2009; Matia-González-Rodríguez-Gabriel-2011 BIOGRID-SMALL-SCALE-STUDIES; Lee-Marcotte-2007 Genetic interactions BIOGRID-SMALL-SCALE-STUDIES; IREF-GRID; IREF-SMALL-SCALE-STUDIES; Lee-Marcotte-2007 Protein interactions; Matia-González-Rodríguez-Gabriel-2011 Huh-O'Shea-2003 |
| *WHI4* | *S. cerevisiae* |  | co-localization | co-expression | Gasch-Brown-2000; Medintz-Thach-2007; Simmons Kovacs-Haase-2012; Huh-O'Shea-2003 |
| *lpd-5* | *C. elegans* | co-expression |  | co-expression | Baugh-Sternberg-2009; Clark-Miska-2010; Honjoh-Nishida-2009; Jans-Meyer-2009; van der Linden-Sengupta-2010 Lewis-Jackson-2009; Phirke-Swoboda-2011 Lewis-Jackson-2009; Von Stetina-Miller-2007 |
| *cisd2* | *Drosophila* |  | physical | phenotype | {Guruharsha, 2011 #5678;Pena-Castillo, 2008 #5721;Eppig, 2007 #5720} |
| *CG17593* | *Drosophila* | co-localization  co-expression  physical | co-expression  physical | predicted | Zhao-Haddad-2010 Chintapalli-Dow-2007 Guruharsha-Artavanis-Tsakonas-2011 B; I2D-BioGRID-Human2Fly; Jordan-Mackay-2007 Guruharsha-Artavanis-Tsakonas-2011 B |
| *CG2135* | *Drosophila* | co-expression | co-expression  co-localization |  | Edwards-Mackay-2006; Innocenti-Morrow-2010; Sørensen-Loeschcke-2005; Qin-Rubin-2007 Chintapalli-Dow-2007 |
| *CG3566* | *Drosophila* | co-expression  physical | co-expression  physical |  | Lundberg-Larsson-2012; Musselman-Baranski-2011 Guruharsha-Artavanis-Tsakonas-2011 A; Wang-Montell-2006 Guruharsha-Artavanis-Tsakonas-2011 B |
| *CG8112* | *Drosophila* |  | co-expression | predicted | Stuart-Kim-2003; Zhao-Haddad-2010 |
| *CG8549* | *Drosophila* |  | co-expression | predicted | I2D-BioGRID-Human2Fly; I2D-BioGRID-Yeast2Fly; Innocenti-Morrow-2010; Lundberg-Larsson-2012 |
| *Sod* | *Drosophila* | co-expression  co-localization |  | co-expression | Lundberg-Larsson-2012; Palanker-Thummel-2009 Chintapalli-Dow-2007; Baker-Russell-2007; Fernández-Ayala-Jacobs-2010 |
| *Ankfy1* | mouse | co-localization | predicted | co-expressed | Siddiqui-Marra-2005; PPI-OPHID; Tarnavski-Izumo-2004 |
| *Gm2a* | mouse |  | co-expressed | co-expressed | Jacobs-Benoist-2010; Zaas-Ginsburg-2010; Zapala-Barlow-2005; Andrechek-Nevins-2009 |
| *PPT2* | mouse |  | co-expressed  phenotype | co-expressed | Boivin-Vidal-2012; Mori-Nevins-2008; PHENOTYPE MGI |
| *HIF1A* | human | co-expression | predicted |  | Peng-Katze-2009; Wu-Stein-2010 |
| *Sec22B* | human | co-expression | co-expression |  | Kang-Willman-2010; Wu-Garvey-2007 |
| *AKR1A1* | human |  | co-expression | co-expression | Wu-Garvey-2007; Gobble-Singer-2011; Hummel-Siebert-2006; Nakayama-Hasegawa-2007; Perou-Botstein-1999 |
| *ATP6V0A1* | human |  | co-localization | co-expression | Gobble-Singer-2011; Radtke-Downing-2009; Johnson-Shoemaker-2003 |
| *DDOST* | human |  | co-expression | physical | BIOGRID-SMALL-SCALE-STUDIES; Kang-Willman-2010; Nakayama-Hasegawa-2007; Ramaswamy-Golub-2001 |
| *FOLR2* | human |  | co-expression | co-expression  co-localization | Nakayama-Hasegawa-2007 Johnson-Shoemaker-2003; Burczynski-Dorner-2006; Wang-Maris-2006 |
| *GRN* | human |  | co-expression | co-expression | Arijs-Rutgeerts-2009; Hummel-Siebert-2006; Nakayama-Hasegawa-2007; Burczynski-Dorner-2006; Gobble-Singer-2011; Nakayama-Hasegawa-2007; Radtke-Downing-2009; Wang-Maris-2006 |
| *RABGGTA* | human |  | co-expression | co-expression | Hummel-Siebert-2006; Wang-Maris-2006 |
| *SOAT1* | human |  | co-expression | predicted | Stuart-Kim-2003; Gobble-Singer-2011; Hummel-Siebert-2006 |
| *STX4* | human |  | co-expression | co-expression | Bild-Nevins-2006 B; Burington-Shaughnessy-2008; Gobble-Singer-2011; Hummel-Siebert-2006; Jones-Libermann-2005; Nakayama-Hasegawa-2007; Rieger-Chu-2004 |
| *ZMPSTE24* | human |  | co-expression | co-expression | Berchtold-Cotman-2008; Bild-Nevins-2006 B; Burington-Shaughnessy-2008; Nakayama-Hasegawa-2007; Wang-Maris-2006 |
